# Supplementary material for: A Bibliometric Analysis of QoR-15 Literature in Perioperative Recovery: Global Research Trends, Collaborations, and Citation Impact
Source: Healthcare (Basel). 2025 Nov 25;13(23):3051. doi: 10.3390/healthcare13233051 (PMC12692248; doi:10.3390/healthcare13233051)
Supplement: Supplementary file 1 [file healthcare-13-03051-s001.zip › healthcare-3906314-supplementary.pdf]

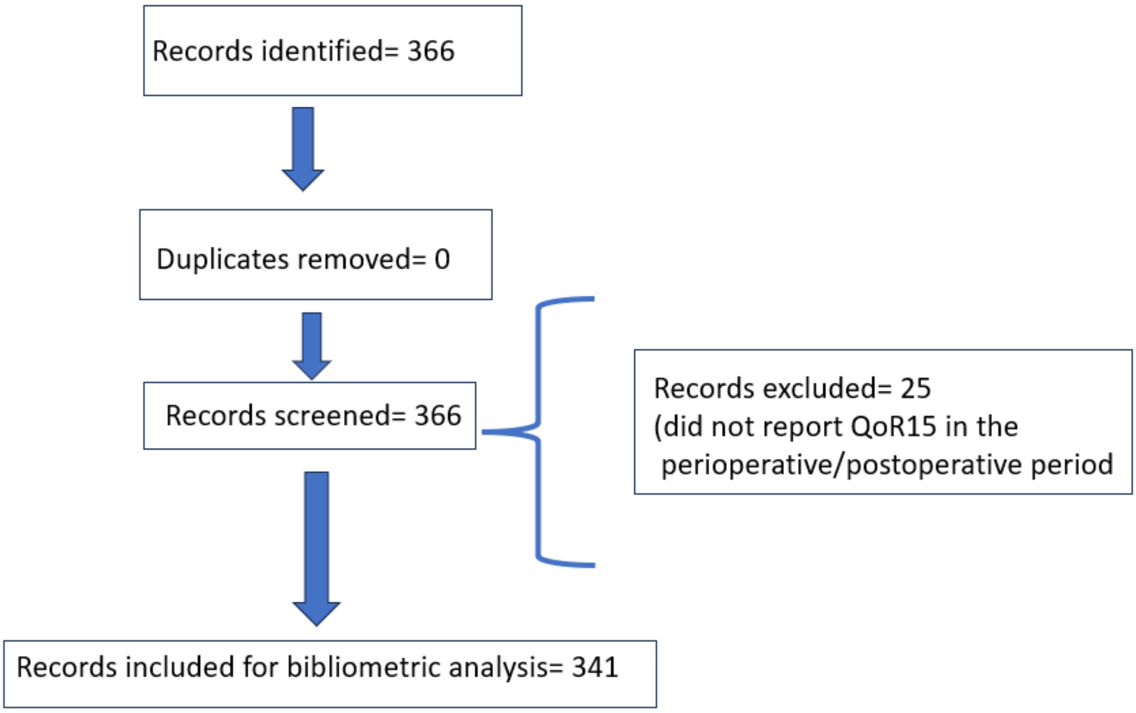

Supplementary Figure S1: PRISMA-style flow diagram showing screening and inclusion of records for the analysis.
